# A Comparative Study of the Lateral Geniculate Body of Rat (*Rattus Norvegicus*), Bat (*Eidolon Helvum*) and Pangolin (*Manis Tricuspis*)

**DOI:** 10.5539/gjhs.v4n4p118

**Published:** 2012-06-12

**Authors:** Temidayo Adeniyi, Ahmad Tijani, Damilare Adekomi, Taiwo Abayomi

**Affiliations:** 1 Department of Anatomy, College of Health Sciences, Osun State University, Osogbo, Nigeria; 2 Department of Anatomy, Faculty of Basic Medical Sciences, University of Ilorin, Ilorin, Nigeria

**Keywords:** lateral geniculate body, lactate dehydrogenase, glucose-6-phosphate dehydrogenase, acid phosphatase, alkaline phosphatase, acetylcholinesterase

## Abstract

In this study, the lateral geniculate bodies (LGB) of rats, bats and pangolins were compared using histological and quantitative histochemical parameters to observe possible modifications that enable these mammals to cope with their habitation particularly with respect to their diet. The study was conducted using ten adult Wistar rats, ten fruit bats and eight pangolins comprising of both sexes. After being sacrificed by cervical dislocation, their skulls were opened using bone forceps to expose the brains. The lateral geniculate bodies were excised from each brain tissue, homogenized and homogenate studied spectrophotometrically for the activities of lactate dehydrogenase (LDH), glucose-6-phosphate dehydrogenase (G-6-PDH), acid phosphatase (ACP), alkaline phosphatase (ALP) and acetylcholinesterase (AChE). The LGB tissue samples meant for histological studies were fixed in 10% formol calcium and processed for paraffin wax embedding. Serial sections of 3μm thickness were stained with Hematoxylin and Eosin (H & E) and Cresyl fast violet (CFV) stains. The stained tissues were studied under the light microscope. Application of one-way ANOVA statistical method showed that there were significant differences (p<0.05) in the activities of LDH, G-6-PDH, ACP, ALP and AChE of the LGB of the three mammals as revealed in the quantitative histochemistry of these enzymes and markers. Histological observations revealed no observable differences in the relative distribution of neurons and their supporting glial cells within the LGB of the three mammalian species. The comparison of the differences observed in the histological and the quantitative histochemical activities in these mammalian species revealed a variation in the visual perception and their individual peculiarities in relation to their mode and pattern of living.

## 1. Introduction

### 1.1 Visual System

Visual perception plays a role in the animal kingdom, most notably for the identification of food sources, suitable habitats, predators, and mate recognition in mating processes, as well as visual functioning to orient animals in their overall ecological surroundings (Cook, 1998). Visual system is the process in which visual perception is achieved. It enables an organism to process visual details, and also enables several non-image forming response functions. It interprets information from visible light to build a representative (picture) of the surrounding world (Schiller, 1986). In mammals, the visual pathways linking the eyes to the brain are those projecting to the lateral geniculate nucleus and superior colliculus (Goodale & Milner, 2004; Kenneth, 2005).

### 1.2 Lateral Geniculate Nucleus

The lateral geniculate nucleus also known as *corpus geniculatum*, is the thalamic relay nucleus for the visual system that lies rostral and lateral to the medial geniculate nucleus, lateral to the *crus cerebi*, and ventral to the *pulvinar* (Williams & Warwick, 1995). The nucleus produces an elevation on the lateral part of the posterior end of the thalamus, and is connected with the superior colliculus by the superior brachium (Carpenter, 1991). The lateral geniculate body is the main thalamic centre for processing visual information by mediating vision and visual perception (Kennedy et al., 1985; Einstein et al., 1987; Jeffery, 1988; Dale et al., 2008).

### 1.3 Mammalia

Mammalia contain a vast diversity of forms ranging from the smallest mammals found among the shrews and bats, to the largest mammal, the blue whale. Mammals have evolved to exploit different ecological niches and life history strategies. Mammals have therefore evolved in various forms in order to perform a wide variety of functions (Vaughan et al., 2000).

The rats, bats and pangolins are nocturnal mammals (Hildebrand & Goslow, 2001). The rat is omnivorous and specializes for life on land (Vaughan, 1986; Feldhamer et al., 1999). Bat (megabats) is frugivorous and arboreal (Kalko et al., 1996; Teeling et al., 2005) while the pangolin is insectivorous and also arboreal (Nowak, 1999).

### 1.4 Significances of Enzymatic Study

G-6-PDH is the first and rate limiting enzyme in the pentose phosphate pathway. G-6-PDH converts nicotinamide adenine dinucleotide phosphate (NADP^+^) into its reduced form, NADPH, and glucose-6-phosphate is then converted into a pentose sugar (ribulose-5-phosphate). The 5-carbon sugar is a precursor of DNA, RNA, and ATP (Peters & Van Noordan, 2009). LDH catalyzes the conversion of pyruvate to lactate with concomitant oxidation of NADH during the last step in anaerobic glycolysis (Coquelle et al., 2007). It converts pyruvate, the final product of glycolysis to lactate when oxygen is absent or in short supply. G-6-PDH and LDH are enzymes of carbohydrate metabolism that are involved in aerobic and anaerobic pathway respectively for ATP production (Sodeinde, 1992).

Acid phosphatase (ACP) is a family of enzymes that belong to the hydrolase class. It possesses the ability to catalyse the hydrolysis of orthophosphate monoesters under acidic conditions (Bull et al., 2002). ACP is the marker enzyme for lysosomes (Pelletier & Novikoff, 1972) since it is found particularly in lysosomes and secretory vesicles. It has been shown that lysosomes, acting presumably via their acid hydrolases, are involved in a variety of cytoplasmic degradative changes during physiological processes (Frost & Brandes, 1967; Anton et al., 1969) especially those involved in neurodegeneration. ACP catalyses and facilitates important physiological changes within cells (Bull et al., 2002). ACP has also been used to monitor cell death and cell lysis (Heryanto et al., 1977; Sarraf & Bowen, 1986; Beem et al., 1987; Yamamoto et al., 2000).

Alkaline phosphatase (ALP) is a group of catalytic proteins found in all body tissues sharing the capacity to hydrolyze phosphate esters in alkaline medium (Zhang et al., 2004; Saini et al., 2005). ALP has been found to act opposite kinases, a function shared with endonucleases. ALP, a membrane biomarker and a regulator of DNA cleavage, mainly facilitates transport across cell membranes, causing the breakdown of ATP to ADP and inorganic phosphate, thereby making free energy available for metabolic processes (Murray et al., 2003).

Acetylcholine (ACh) is a neurotransmitter common to many synapses throughout mammalian nervous system ranging from the diffused system in the brain to the neuromuscular junctions. Acetylcholinesterase (AChE) is one of the most crucial enzymes for nerve response and function (Seeger et al., 2008). AChE catalyses the hydrolysis of acetylcholinesters with a relative specificity for acetylcholine mainly in diffused neurons and cholinergic neuromuscular junctions (Seeger et al., 2008; Mosby, 2009). Acetylcholinesterase (AChE) degrades acetylcholine thereby producing choline and an acetate group. It serves to terminate synaptic transmission (Dale et al., 2008). Quantitative histochemistry is a potent method for detecting localized changes in AChE content (Biegon et al., 1986). AChE is therefore, a measure of the cholinergic neuronal activity.

This research work was therefore designed to compare differences in the histological and quantitative histochemical activities in the LGB of the mammalian species that enable them adapt and cope with their habitat despite their varying feeding habits, mode of lives and habitat differences.

## 2. Materials and Methods

### 2.1 Experimental Animals

Ten adult Wistar rats (*Rattus norvagicus*), ten fruit bats (*Eidolon helvum*), and eight pangolins (*Manis tricuspis*) of both sexes were used for this comparative study. The adult Wistar rats were obtained from the animal holdings of the department of Anatomy of the University of Ilorin and sacrificed shortly after procurement. The bats were curled down from their roosting colony with the assistance of experts who possess state permit at the bats colony at the Flower Garden area of Government Reserve Area (GRA), Ilorin and sacrificed shortly thereafter. The pangolins were procured from Asejire, a village in the North West Area of Osun State and sacrificed before dark the same day. All experimental procedures followed the recommendations provided in the “Guide for the Care and Use of Laboratory Animals” prepared by the National Academy of Sciences and published by the National Institute of Health (NIH, 1985). The G6-PDH, LDH and ACP kits were purchased from Randox laboratory limited, UK, ALP from Quimica Clinica Aplicada’s, Spain and AChE kit from AGAPPE DIAGNOSTIC LTD, UK.

### 2.2 Methods

The animals were sacrificed by cervical dislocation. The skulls of the sacrificed animals were opened using bone forceps to expose the brain. The lateral geniculate bodies were located via tracing the optic tract to the optic chiasma and excised with the aid of the Atlas of the Rat Brain in Sterotaxic Co-ordinates (Paxinos & Watson, 2004). The specimens for routine histological investigations were fixed in formol calcium and processed for paraffin wax embedding. Serial sections of 3µm thickness were stained with Haematoxylin and Eosin and Cresyl Fast Violet. Those for quantitative histochemical enzyme studies were preserved separately in cold 0.25M sucrose (Isotonic solution) and were homogenized with Polter-Elvhjem homogenizer.

The homogenates were centrifuged at 5000rpm for 10 minutes. The supernatants were immediately stored in the freezer (-20°C) and assayed within 48 hours. Through spectrophotometry, the activities of G-6-PDH, LDH, ACP, ALP and AChE were determined in the homogenate by the methods of Lohr and Waller (1974), Wei Bhaar (1975), Babson et al. (1966) and Knedel & Bottger, (1967) respectively.

#### 2.2.1 Statistical Analysis

Values were reported as mean ± SEM (standard error of the mean). Significance was determined statistically by application of one-way analysis of variance (ANOVA) using statistical software SPSS version 17 at 95% confidence interval. Differences between means were considered statistically significant at P<0.05.

## 3. Results

### 3.1 Histological Observations

**Figure 1:**
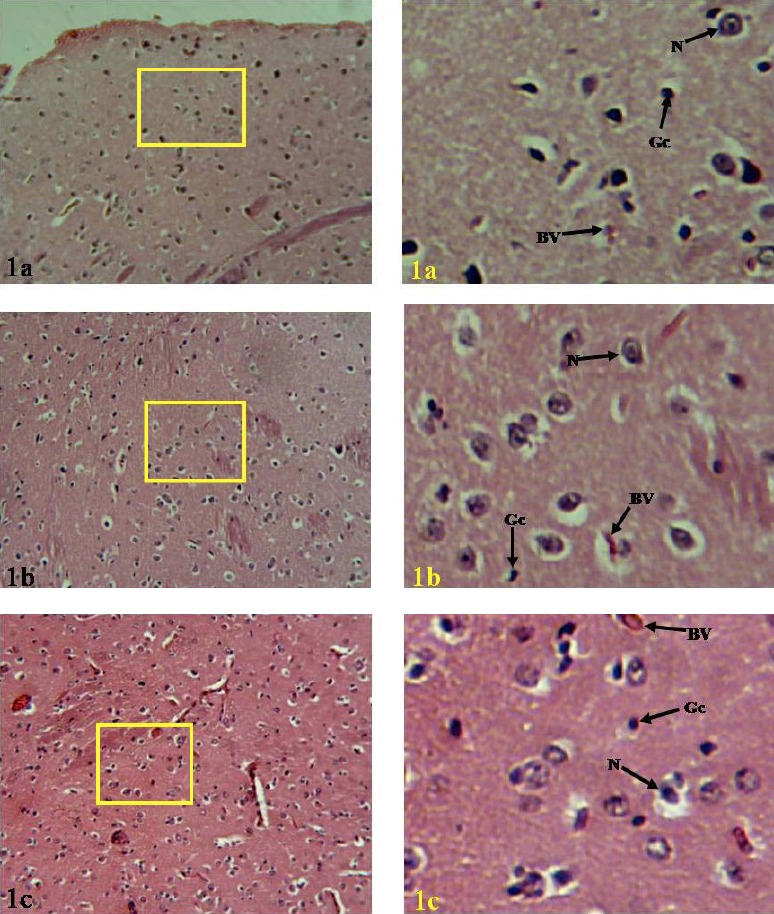
Photomicregraphs of the lateral geniculate body of Rat(1a), Bat(1b) and Pangolin(1c) Legends: BV=Blood Vessel, N=Neuron, Gc=Glial cell. (H&E, ×180(left), ×720(right)) Note: the panoramic view of the yellow bordered region is presented on the right

It was observed in the sections stained in H & E and demonstrated in the photomicrographs labeled Figure 1, that the neurons were equally and evenly distributed within their LGBs. In the rats, neurons of the LGB were seen to be orientated in this laminated structure with the orientation of the fibres seen to be different. The orientation of the cells is directed centrally towards the laminae such that the cells were seen to have projections that are directed at right angle and divide rapidly towards these laminae. In the Bat, uniform neuronal types were seen as different from the distinct neuron types observed in the rats, and the fibres are multidirectional from distinct cell compartments. The cells are large in size compared to those seen in the rat LGB. In the Pangolin LGB, the cells were seen in units of two to three cells. This is also suspected to be multi adapted cells and the fibres are not entirely directed towards a single direction. The region is also highly vascularized compared with the rats and bats.

**Figure 2:**
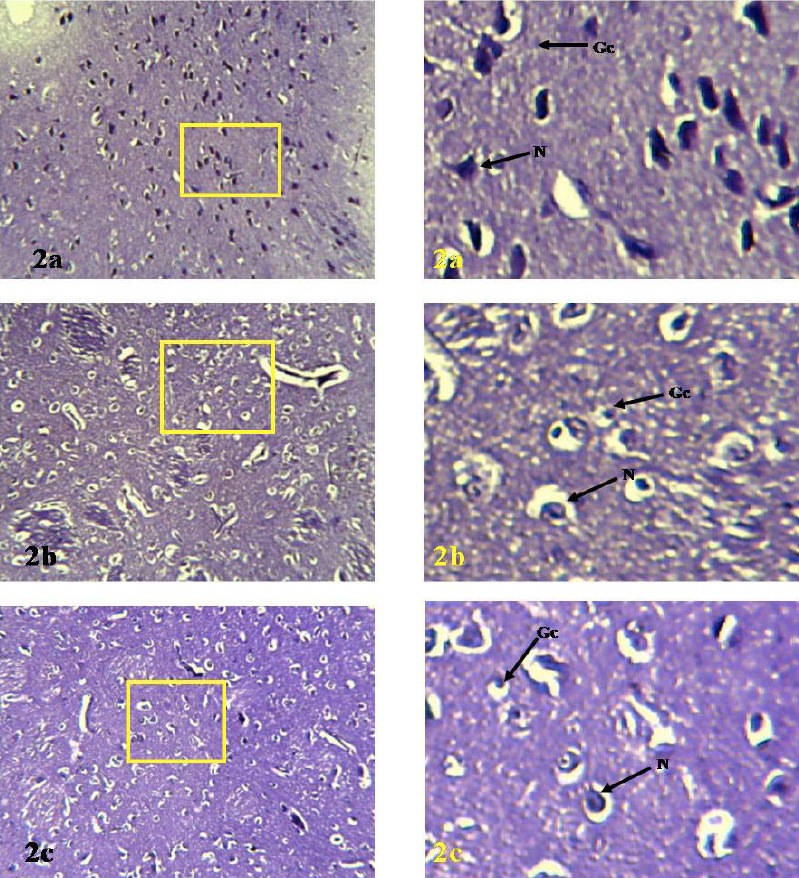
Photomicrographs of the lateral geniculate body of Rat(1a), Bat(1b) and Pangolin(1c) Legends: N=Neuron, Gc=Glial cell. (H&E, ×180(left), ×720(right)) Note: the panoramic view of the yellow bordered region is presented on the right

Using Cresyl Fast Violet (CFV) staining technique to compare the abundance of Nissl substance of the mammalian species as demonstrated in the photomicrographs labelled Figure 2, the intensively stained Nissl substances are apparent in all the animals; the nuclei in the cells are also visible.

### 3.2 Statistics and Data Analysis

Result obtained from the present study showed significant differences in the activities of the enzymes studied using quantitative histochemistry (Table 1).

**Table 1:** Enzyme activities in the lateral geniculate bodies of the three mammals (IU/L)

Animals	G-6-PDH	LDH	ACP	ALP	AChE
Rat	1271.33±21.21*	1399.66±4.91*	10.60±0.36*	3.16±0.17*	534.82±4.66*
Bat	681.33±21.17*	819.00±4.72*	13.50±0.77*	4.56±0.20*	225.66±6.38*
Pangolin	842.00±14.01*	479.66±17.57*	7.76±0.38*	6.16±0.13*	128.00±1.73*

* level of significance (p<0.05) (G-6-PDH p=0.000) (LDH p=0.002) (ACP=0.001) (ALP=0.000) (AchE p=0.000)

## 4. Discussion

Comparatively, the histological observations of the lateral geniculate body (LGB) of the three mammalian species stained with H & E method illustrated in Figure 1 showed no observable differences in the relative distribution of the neurons within the LGB. The micrographs showed that the neurons and glial cells are equally and evenly distributed within their LGBs. These observations strongly suggested that all the mammalian species employed the use of their LGB for visual perception since the lateral geniculate body is the main thalamic centre for processing visual information by mediating vision and visual perception (Kennedy et al., 1985; Einstein et al., 1987, Jeffery, 1988, Dale et al., 2008).

Nissl bodies are strongly basophilic inclusions found in the cell bodies of neurons. These granules are rough endoplasmic reticulum with free ribosomes and are the sites of protein synthesis. Increased Nissl substances as evidenced in Figure 2 in the LGB of the rat and pangolin, indicates that the cells are highly active in protein synthesis while those of the bat are moderate.

Cortical Structures including lateral geniculate body have higher glucose utilization than other anatomical structures (Siesjo, 1978). Glucose utilization of these structures is achieved through carbohydrate metabolism. Glucose-6-phosphate dehydrogenase (G-6-PDH) and lactate dehydrogenase (LDH) are enzymes of carbohydrate metabolism that are involved in aerobic and anaerobic pathway respectively for ATP production (Sodeinde, 1992).

The measure of the quantitative histochemical activity of G-6-PDH in LGB in the three mammalian species showed significant variations. LGB of rat employed the largest amount of G-6-PDH activity; hence is the most likely using the largest quantity of energy to mediate vision and visual perception. This is followed by pangolin while the bat had the least.

In overexertion conditions when oxygen is absent or in short supply to cope with energy demands of the LGB, LDH comes in to play in energy production. From the result obtained, LDH in rat LGB had the highest the quantitative histochemical activity, followed by the bat and the least was observed in the pangolin. The rat LGB utilizes the highest base on its energy demand to cope with lifestyle. Bat engages in true flight and employs an alternative pathway for its metabolism. This suggests why bat have a higher activity level than pangolin. A closer look at the G-6-PDH:LDH in examining carbohydrate metabolism as a balance between aerobic and anaerobic metabolism shows;

The ratio of the activities of G-6-PDH:LDH in rats indicate that the anaerobic system is more active than the aerobic system, while the same was observed in the Bats with the levels of LDH being higher than those of G-6-PDH indicating a negative shift in oxygen usage for metabolism, while a positive shift was seen in the pangolin.

The measure of ACP activity was found to be higher in bat LGB, followed by rat and pangolin has the least. The higher ACP activity in bat LGB may indicate higher amount of unused substances in form of precursors of neurotransmitters within the lateral geniculate nuclei mediating vision and visual perception. It also help to “mop up” the phosphate ions produced as a result of ATP hydrolysis.

The measure of ALP activity was however found to be higher in the LGB of pangolin followed by bat and the least being rat. Pangolins are more likely generating more free energy through ALP to compliment for their LGB metabolic process. ALP mainly facilitates transport across cell membranes, causing the breakdown of ATP to ADP and inorganic phosphate, thereby making free energy available for metabolic process (Murray *et al.*, 2003).

The measure of the cholinergic neurons was compared in the LGB of the mammalian species. It was found that the rat LGB uses higher amount of AChE; hence employs most of this neurotransmitter to mediate neural impulses, followed by bat and then pangolin. AChE is the neurotransmitter common to many synapses throughout mammalian nervous systems. Depending on the type and function of a particular neuron, neurotransmitters may cause or inhibit the transmission of neural impulses. The physical and neurochemical characteristics of each synapse determine the strength and polarity of the new input signal.

The measure of the histological observations and quantitative histochemistry in the lateral geniculate body of the mammalian species confirms that the variations observed were due to their individual peculiarities. The feeding habits, lifestyle as well as the nature of their habitat would account for this. While rats are active during the day and night with higher mobility, pangolins are only active at night. However, being an insectivore, they require highly effective vision to identify and contrast food which could be quite small, although, olfactory communication also plays a significant role (Feldhamer et al., 2007). The bats also are active during the day and night. They feed on fruits and depend on the combined use of echolocation and olfactory clues to find ripe fruits hidden and nestled among leaves (Laska, 1990; Hessel & Schmidt, 1994; Rieger & Jacob, 1988; Kalko & Codon, 1998; Thies et al., 1998; Korine et al., 2000; Mikich et al., 2003).

## 5. Conclusion

The histological and quantitative histochemical results revealed a relationship between the visual perception and specie peculiarities of the mammalian species in relation to their mode and pattern of living. This however maybe suggested as the reason for the difference in the feeding and habitat pattern of the studied mammalian species.

## References

[ref1] Anton E, Brandes D, Barnard S (1969). Lysosomes in uterine involution: distribution of acid hydrolases in luminal epithelium. Anat. Rec.

[ref2] Babson L. A, Greeley S. J, Coleman C. M, Philips G. D (1966). Serum alka-line phosphatase determination. Clinical Chemistry.

[ref3] Beem E. P, Hillebrand M. J, Benckhuijsen C, Overdijk B (1987). Origin of the increased activity of beta glucuronidase in the soluble fraction of rat mammary tumors during ovariectomy-induced regression. Cancer Res.

[ref4] Biegon A, Greenberger V, Segal M (1986). Quantitative histochemistry of brain acetylcholinesterase and learning rate in the aged rat. Neurobiol. Aging.

[ref5] Bull H, Murray P. G, Thomas D, Fraser A. M, Nelson P. N (2002). Acid Phosphatases. Journal of Clinical Pathology. Mol. Pathol.

[ref6] Carpenter M. B (1991). Core text of Neuroanatomy.

[ref7] Cook R. G, Greenberg G, Haraway M (1998). Visual perception. Encyclopedia of comparative psychology.

[ref8] Coquelle N, Fioravanti E, Weik M, Vellieux F, Madern D (2007). Activity, Stability and Structural Studies of Lactate Dehydrogenases Adapted to Extreme Thermal Environments. J. Mol. Biol.

[ref9] Dale P, George J. A, David F, William C. H, Anthony-Samuel L, James O. M, Leonard E. W (2008). Neuroscience.

[ref10] Einstein G, Davis T. L, Sterling P (1987). Pattern of lateral geniculate synapse on neuron somata in layer IV of the cat striate cortex. J. Comp. Neurol.

[ref11] Feldhamer G. A, Lee C. D, Stephen H. V, Joseph F. M (2007). Mammalogy, Adaptation, Diversity and Ecology.

[ref12] Feldhammer G. A, Drickamer L. C, Vessey S. H, Merrilt J. F (1999). Mammalogy. Adaptation, Diversity and Ecology.

[ref13] Frost J. L, Brandes D (1967). Nonspecific esterases in rat prostatic epithelial cells. J. Histochem. Cytochem.

[ref14] Goodale M, Milner D (2004). Sight unseen.

[ref15] Heryanto B, Yoshimura Y, Tamura T, Okamoto T (1977). Involvement of apoptosis and lysosomal hydrolase activity in the oviducal regression during induced molting in chickens: a cytochemical study for end labeling of fragmented DNA and acid phosphatase. Poult. Sci. J.

[ref16] Hessel K, Schmidt U (1994). Multimodal orientation in Carollia perspicillata (Phyllostomidae Folia Zool).

[ref17] Hildebrand M, Goslow G. E (2001). Analysis of vertebrate structure.

[ref18] Jeffery G (1988). Shifting retinal maps in the development of the lateral geniculate nucleus. Dev. Brain Res.

[ref19] Kalko E. K, Condon M (1998). Echolocation, olfaction and fruit display: How bat find fruit of flagella chorous cucurbits. Funct. Ecol.

[ref20] Kalko E. K. V, Herre E. A, Handly C. O (1996). Relation of fig fruit characteristics to fruit-eating bats in the New and Old world tropics. J. Biogeography.

[ref21] Kennedy H, Bullier J, Dehay C (1985). Cytochrome oxidase activity in the striate cortex and lateral geniculate nucleus of the newborn and adult macaque monkey. Exp. Brain Res.

[ref22] Kenneth L. G (2005). Binocular visual responses in cells of the rat dLGN. J. Physiol.

[ref23] Knedel M, Böttger R (1967). A kinetic method for determination of the activity of pseudocholinesterase (acylcholine acyl-hydrolase 3.1.1.8.). Klin Wochenschr.

[ref24] Korine C, Kalko E. O, Herre E. A (2000). Fruit characteristics and factors affecting fruit removal in a Panamanian community of strangler figs. Oecologia.

[ref25] Laska M (1990). Olfactory Sensitivity to food odour components in the short-tailed fruit-bat, Cocrolliata perspispicillata (Phyllostomatidae, Chiroptera). J. Comp. Physiol. A.

[ref26] Lohr G. W, Waller H. D (1974). Glucose-6-Phosphate Dehydrogenase. Methods of Enzymatic Analysis.

[ref27] Mikich S. B, Bianconi G. V, Maia B, Teixera S. D (2003). Attraction of the fruit-eating bat Carollia perspicillata to Piper qaudichaudanium essiential oil. J. Chem. Ecol.

[ref28] Mosby E (2009). Mosby’s medical dictionary.

[ref29] Murray R. K, Granner D. K, Mayes P. A, Rodwell V. W (2003). Harper’s illustrated biochemistry.

[ref30] National Institutes of Health (1985). Guide for the Care and Use of Laboratory Animals: DHEW Publication (NIH), revised.

[ref31] Nowak R. M (1999). Walker’s Mammals of the World Volume II.

[ref32] Paxinos G, Watson C (2004). The rat brain in sterotaxic co-ordinates.

[ref33] Pelletier G, Novikoff A. B (1972). Localization of phosphatase activities in the rat anterior pituitary gland. J. Histochem. Cytochem.

[ref34] Peters A. L, Van Noorden C. J. F (2009). Glucose-6-phosphate Dehydrogenase Deficiency and Malaria: Cytochemical Detection of Heterozygous G6PD Deficiency in Women. J. Histochem. Cytochem.

[ref35] Rieger J. E, Jacob E. M (1988). The use of olfaction in food location by frugivorous bats. Biotrop.

[ref36] Saini D, Kala M, Jain V, Sinha S (2005). Targeting the active site of the placental isozyme of alkaline phosphatase by phage-displayed scFv antibodies selected by a specific uncompetitive inhibitor. BMC Biotech.

[ref37] Sarraf C. E, Bowen I. D (1986). Kinetic studies on a murine sarcoma and an analysis of apoptosis. Br. J. Cancer.

[ref38] Schiller P. H (1986). The central visual system. Vision Research.

[ref39] Seeger G, Aldrich S, Bartos J (2008). Acetylcholinesterase: function, struction, and inhibition. Molecule description.

[ref40] Siesjo B. K (1978). Utilization of Substrates by brain tissues. Brain energy metabolism.

[ref41] Sodeinde O (1992). Glucose-6-phosphate dehydrogenase deficiency. Brailleire’s Clinical Haematology.

[ref42] Teeling E, Springer M, Madsen O, Bates P, O’Brien S, Murphy W (2005). A molecular phylogeny for bat illuminates biogeography and the fossil record. Science.

[ref43] Thies W, Kalko E. K. V, Schnitzler H. U (1998). The role of echolocation and olfaction in two Neotropical fruit-eating bats, Carllia Perspicillata and C. Castame, feeding on Piper. Behav. Ecol. Sciol.

[ref44] Vaughan T (1986). Mammalogy.

[ref45] Vaughan T, Ryan J, Czaplewski N (2000). Mammalogy.

[ref46] Wei Bhaar D (1975). Med. Weit.

[ref47] Williams P. L, Warwick R (1995). Gray’s anatomy.

[ref48] Yamamoto S, Sawada K, Shimomura H, Kawamura K, James T. N (2000). On the nature of cell death during remodeling of hypertrophied human myocardium. J. Mol. Cell Cardiol.

[ref49] Zhang L, Buchet R, Azzar G (2004). Phosphate binding in the active site of alkaline phosphatase and the interactions of 2- nitrosoacetophenone with alkaline phosphatase-induced small structural changes. Biophys. J.

